# Molecular diagnostic and predictive tests in the evolution of chronic hepatitis C anti-viral therapies

**DOI:** 10.1186/1471-2334-12-S2-S8

**Published:** 2012-11-12

**Authors:** Giuseppe Colucci

**Affiliations:** 1 Roche Diagnostics, GCS, CH 6343 Rotkreuz, Switzerland

## Abstract

Since the discovery of HCV, polymerase chain reaction (PCR) has significantly contributed to the understanding of the virus life cycle and its replicative kinetics during anti-viral therapy. Parallel to the progression of dual and triple combination treatment, real-time PCR molecular tests have constantly improved in their ability to monitor viral load and drive personalized management schedules. The current sensitivity, accuracy and dynamic range of the available assays fulfil the requirement of “companion diagnostics” and support the development of new directly acting antiviral (DAA)-based regimens.

## Introduction

The development of polymerase chain reaction (PCR) in the eighties was instrumental, a few years later, to the discovery of HCV, the first infectious disease agent identified by a reverse genetic approach. Using a library of primers and concentrated plasma from individuals with non-A, non-B hepatitis, M. Houghton et al. amplified and identified the first HCV sub-genomic fragments whose products were specifically reactive with circulating antibodies from infected patients [1]. Proteins derived from the initial HCV clones served as target antigens in the first serological assay and, once validated in large cohorts of patients with non-A, non-B hepatitis, they were further developed into standardized immunoassays for screening blood donations and blood derived products to prevent transmission.

## Development of molecular methods in the diagnosis of HCV

**Figure 1:**
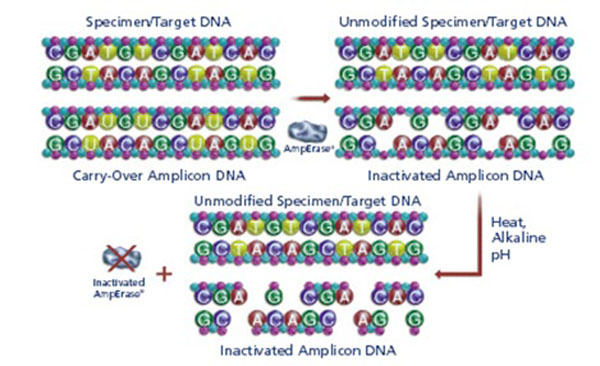
Amperase® mediated selected inactivation of amplicons from previous reactions. Amplicons are synthetized during PCR using dUTP instead of dATP and made susceptible to cleavage by Amperase® which has no effect on sample, native DNA.

The first PCR-based tests were developed as confirmatory assays for seropositive individuals and to stage the replicative activity of the infection. Two major technological innovations enabled the broad uptake of these initial PCR tests: (i) the discovery of thermo-stable enzymes with both reverse transcription and DNA polymerase activity (Tth polymerase); (ii) the introduction of uracyl-N-glycosilase (Amperase®) [2,3]. The former allowed for a single tube-single reaction PCR workflow, while the latter eliminated carry over contamination by selective inactivation of amplicons from previous reactions (Figure 1).

The evidence available at that time on the clinical utility of viral load in the area of HIV/AIDS suggested similar indications also for the management of chronic hepatitis C and led to the development of quantitative PCR tests based on end-point dilution. These provided the initial tools to investigate the association between viral load and disease progression, as well as response to therapy.

Different response profiles were identified during interferon (IFN) based therapies and a first, viral kinetics based, predictive criterion was introduced in clinical practise which identified, at week 12 on treatment, patients with no probability of sustained virological response (SVR). SVR is defined by a negative HCV RNA test 6 months after end of treatment and indicate virus eradication. A decrease of less than 2 logarithms at week 12, the threshold required for the early virological response (EVR), was found to have a very high negative predictive value and was proposed and validated as a “stopping rule” for treatment discontinuation.

**Figure 2:**
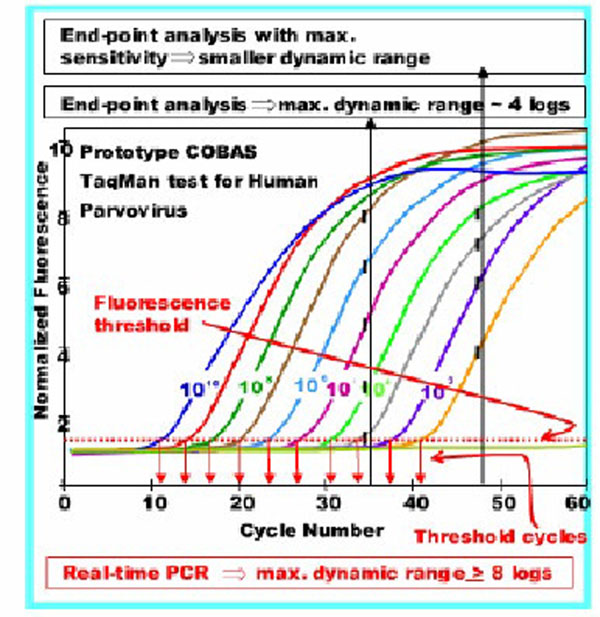
Progressive increase in sensitivity and dynamic ranges when proceeding from end point to real-time PCR. The latter allowed for a linear range increase of up to 4 logarithms.

Only after the development of the real-time, Taqman PCR technique, it was possible to introduce truly quantitative molecular test and proceed with a thorough assessment of viral load kinetics and its clinical correlates. As previously reported [4], the Taqman method is based on dual fluorescent dye-labeled probes with a reporter and a quencher fluorochrome at the 5´and 3´regions. In the probe native state, the energy emitted by the reporter dye is absorbed by the quencher, which prevents light emission. During amplification, the probe hybridizes to its complementary target DNA and is cleaved by the exonuclease activity of the DNA Polymerase. The two dyes are released, quenching no longer occurs and a fluorescent signal is produced whose intensity is proportional to the amount of the sample target DNA or RNA. Compared to the initial end-point PCR based test, real-time assays have much broader linear ranges and higher accuracy (Figure 2).

Using these tests, an additional, important viral load parameter was identified at week 4 on treatment, the rapid virological response (RVR), characterized by undetectable HCV RNA, with a high positive predictive value for SVR so as to serve as a main driver for treatment individualization.

The recent development of DAA and the approval of the first, boceprevir- and telaprevir-based triple-therapy regimes, have brought new hopes for higher virus eradication rate across different disease settings. This calls for even more accurate and sensitive quantitative HCV RNA tests able to monitor faster virus kinetics and promptly detect treatment failures. Indeed, subtle differences between a HCV RNA negative (reported as target not detected) and a borderline HCV RNA positive result (reported as detected <15 IU/ml), below the limit of detection (LOD) of the most widely used real-time PCR tests, were found to influence the RVR rate and potentially lead to more relapses.

**Figure 3:**
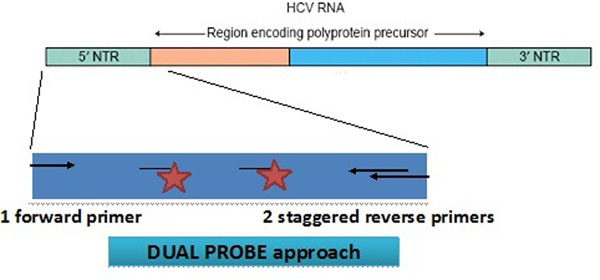
Dual probe design of the Cobas® Ampliprep™/Cobas® Taqman™ HCV 2.0 test second version. The introduction of a second labelled probe and an additional reverse primers increased mismatchs tolerance and assay’s accuracy.

In this respect, we have recently introduced a new version of the Cobas® Ampliprep™/Cobas® Taqman™ HCV 2.0 test, a fully automated real-time PCR assay, which has a novel dual-probe design which minimizes the impact of possible sequence mismatches (Figure 3). Similar to the dual target approached followed in the development of the Cobas® Ampliprep™/Cobas® Taqman™ HIV 2.0 test, where both the LTR and the gag genes are amplified, the HCV 2.0 assay has an increase genotypes inclusivity and better coverage of minor variants as well as of new strains and mutants that may emerge in the future. This new configuration also allowed for setting a unique threshold for both the LOD and the limit of quantification (LOQ) to improve the interpretation of results and increase precision at the lower end of the linear range [5,6].

## Conclusions

With new viral load tests acting as “companion diagnostic”, the clinical development and applications of the new generation DAA can be fully supported for a more effective management of chronic hepatitis C and its long-term complications.

## Competing interests

The author is employed by Roche Diagnostics.

## Declarations

Publication of this supplement was partly supported by an unrestricted grant provided by Roche. The articles were independently prepared by the authors with no input from Roche. Roche were not involved in selecting the articles for the supplement.
